# Foliar-Applied Selenium–Zinc Nanocomposite Drives Synergistic Effects on Se/Zn Accumulation in *Brassica chinensis* L.

**DOI:** 10.3390/nano16010056

**Published:** 2025-12-31

**Authors:** Mengna Tao, Yusong Yao, Lian Zhang, Jie Zeng, Bingxu Cheng, Chuanxi Wang

**Affiliations:** Institute of Environmental Processes and Pollution Control, School of Environment and Ecology, Jiangnan University, Wuxi 214122, China

**Keywords:** biofortification, metabolic reprogramming, nutrient co-accumulation, transporter regulation, rhizosphere microbiota

## Abstract

Micronutrient malnutrition persists as a global health burden, while conventional biofortification approaches suffer from low efficiency and environmental trade-offs. This study aimed to develop and evaluate a foliar-applied selenium–zinc nanocomposite (Nano-ZSe, a mixture of zinc ionic fertilizer and nano selenium) for synergistic Se/Zn co-biofortification in *Brassica chinensis* L., using a controlled pot experiment that integrated physiological, metabolic, molecular, and rhizosphere analyses. Application of Nano-ZSe at 0.18 mg·kg^−1^ (Based on soil weight) not only increased shoot biomass by 28.4% but also elevated Se and Zn concentrations in edible tissues by 7.00- and 1.66-fold (within the safe limits established for human consumption), respectively, compared to the control. Mechanistically, Nano-ZSe reprogrammed the ascorbate-glutathione redox system and redirected carbon flux through the tricarboxylic acid cycle, suppressing acetyl-CoA biosynthesis and reducing abscisic acid accumulation. This metabolic rewiring promoted stomatal opening, thereby enhancing foliar nutrient uptake. Simultaneously, Nano-ZSe triggered the coordinated upregulation of *BcSultr1;1* (a sulfate/selenium transporter) and *BcZIP4* (a Zn^2+^ transporter), enabling synchronized translocation and the tissue-level co-accumulation of Se and Zn. Beyond plant physiology, Nano-ZSe improved soil physicochemical properties, enriched rhizosphere microbial diversity, and increased crop yield and economic returns. Collectively, this work demonstrates that nano-enabled dual-nutrient delivery systems can bridge nutritional and agronomic objectives through integrated physiological, molecular, and rhizosphere-mediated mechanisms, offering a scalable and environmentally sustainable pathway toward functional food production and the mitigation of hidden hunger.

## 1. Introduction

Global food systems are increasingly strained by the dual imperative of sustaining yield under climate pressure while delivering nutrient-dense produce to counteract widespread micronutrient malnutrition [1,2]. Conventional agriculture, optimized for caloric output rather than nutritional quality, has proven insufficient in addressing “hidden hunger”—a condition affecting billions due to inadequate dietary intake of essential trace elements such as zinc (Zn) and selenium (Se) [3]. Although Zn and Se are critical for human immune function, metabolic regulation, and antioxidant defense, their concentrations in edible crops remain low in many agroecosystems, particularly where soils are inherently deficient in bioavailable forms [4,5]. Selenium deficiency in the diet represents a significant global public health problem and may lead to serious health consequences. It is estimated that inadequate selenium intake affects up to one billion people worldwide [6]. Similarly, Zn deficiency affects approximately 17.3% of people worldwide, with insufficient intake linked to over 400,000 annual deaths in children under five from infectious diseases [7,8]. These challenges underscore the urgent need for scalable strategies that enhance the nutritional value of food at the source through crop biofortification [9]. Dietary intake of Se- and Zn-biofortified crops represents one of the most sustainable pathways to bridge this nutrient gap [10]. Traditional approaches using ionic fertilizers (e.g., zinc sulfate, sodium selenite) have shown modest success in staple crops such as wheat, rice, and strawberry [11,12]. However, the agronomic efficacy of conventional biofortification is severely constrained when micronutrients are applied individually in soluble ionic forms, due to low bioavailability resulting from soil fixation, leaching, and redox-driven immobilization, leading to poor nutrient use efficiency [13,14,15]. Moreover, repeated single-element applications can disrupt soil chemistry and alter microbial community structure over time [16,17]. In contrast, engineered nanomaterials (ENMs) offer a transformative alternative: their high surface-to-volume ratio, tunable dissolution kinetics, and capacity for foliar uptake enable more efficient and targeted nutrient delivery [18,19,20]. Nanofertilizers have been shown to increase nutrient bioavailability by up to 30% compared to conventional sources across diverse cropping systems [21]. Beyond enhanced uptake, and as noted above, the use of single-element fertilizers entails significant limitations, whereas recent studies have demonstrated that synergistic co-accumulation of multiple micronutrients is not only feasible but advantageous [22,23,24]. For instance, ENMs can promote multi-nutrient co-accumulation by modulating the expression of metal transporter genes (e.g., ZIP, NRAMP), stimulating root exudation of organic acids, and enriching beneficial rhizosphere microbiota that facilitate nutrient solubilization [22,23]. Carbon dots (CDs) elevated Zn concentrations in plant tissues by 41.2% through coordinated upregulation of Zn^2+^ transporters, enhanced photosynthesis, and the recruitment of plant growth-promoting rhizobacteria [24]. These findings highlight the potential of nano-enabled platforms to achieve synergistic co-biofortification, yet a critical knowledge gap remains: can a single nanocomposite simultaneously and efficiently deliver both Se and Zn to edible tissues?

Interestingly, co-application of sodium selenite and Zn^2+^ fertilizers may lead to ionic antagonism and thus hinders simultaneous Se and Zn enrichment [25]. Se ENMs have emerged as a leading candidate for Se biofortification due to their low phytotoxicity, high biocompatibility, and superior bioavailability relative to ionic Se species [26,27,28]. Foliar-applied Se ENMs have been shown to enhance photosynthetic efficiency in lettuce, increase grain yield in rice, and improve fruit quality in tomato by modulating sugar-acid balance and antioxidant content [29,30]. Notably, Se ENM application has also been associated with concurrent Zn accumulation in plum, pomegranate, and *Brassica chinensis* L. [31,32,33], suggesting cross-element interactions that may enable dual enrichment. For example, Zhou et al. demonstrated that foliar application of nano-selenium (nano-Se) not only significantly enhanced the selenium content in plum leaves (by 11.4-fold compared to the control) but also synergistically increased the accumulation of zinc (Zn), with Zn levels rising by 30.9% [31]. Zahedi et al. found that foliar application of selenium nanoparticles (N-Se) at a concentration of 2 μM significantly increased not only the selenium content in pomegranate leaves but also enhanced the zinc (Zn) content [32]. Similarly, Zhu et al. also illustrated that nano-selenium can simultaneously enhance selenium and zinc accumulation, thereby boosting the mineral nutrient profile of the vegetable [33]. However, the physiological and molecular mechanisms underpinning this synergy, particularly the roles of stomatal regulation, redox metabolism, and transporter coordination, remain poorly understood. *Brassica chinensis* L. (*B. chinensis*), serves as an ideal model for such investigations due to its rapid growth, high biomass, and inherent capacity for mineral accumulation, making it highly responsive to nano-agronomic interventions [34].

Here, we design and evaluate a foliar-applied selenium–zinc nanocomposite (Nano-ZSe) to achieve synergistic co-biofortification in *B. chinensis*. Therefore, this study specifically aims to (1) evaluate the efficacy of a foliar-applied selenium–zinc nanocomposite (Nano-ZSe) in achieving safe and synergistic co-accumulation of Se and Zn in *Brassica chinensis* L.; (2) elucidate the metabolic and molecular mechanisms—particularly redox regulation, carbon flux redirection, and coordinated transporter expression—that underpin enhanced foliar uptake and tissue-level co-biofortification; and (3) assess the cascading effects of Nano-ZSe on rhizosphere microbiota composition and soil health, thereby linking plant physiological responses to belowground ecosystem functions. By bridging nano-delivery design with plant system responses, this work advances a scalable, environmentally conscious strategy to produce multifunctional foods that address hidden hunger without compromising ecosystem integrity.

## 2. Materials and Methods

### 2.1. Synthesis and Characterization of Nano-ZSe

Nano-ZSe was synthesized via a one-pot wet-chemical reduction method. Selenium dioxide (SeO_2_, analytical reagent grade) served as the selenium precursor, with ascorbic acid (AR) as the reducing agent and polyvinylpyrrolidone (PVP, AR) as the stabilizer. The reagents were sequentially dissolved in deionized water under continuous magnetic stirring to obtain a homogeneous solution. Following the formation of a stable Se ENMs suspension, a commercial Zn-based fertilizer (Zn concentration: 160 g·L^−1^) was added dropwise under vigorous mechanical agitation to achieve physical mix. The resulting colloidal suspension was centrifuged, and the precipitate was collected, repeatedly washed with deionized water to remove residual species, and subsequently lyophilized to yield the Nano-ZSe powder.

For morphological characterization, the as-synthesized Nano-ZSe was redispersed in deionized water by ultrasonication to form a stable suspension. A droplet of this suspension was deposited onto a carbon-coated copper grid and air-dried at room temperature. Particle morphology, nanostructure, and size distribution were analyzed by high-resolution transmission electron microscopy (TEM, JEM-2100, Nippon Electronics Co., Ltd., Tokyo, Japan). At least 200 individual particles were measured from TEM micrographs using ImageJ 1.54g software to determine the mean diameter and polydispersity index. Surface elemental composition and chemical states were further characterized by X-ray photoelectron spectroscopy (XPS, K-Alpha, Thermo Fisher, Birmingham, UK).

### 2.2. B. chinensis Cultivation and Measurement of Physiological Parameters

#### 2.2.1. *B. chinensis* Cultivation

The experiment was conducted with *B. chinensis* seeds obtained from Nongdianzan Company (Shenzhen, China). Seeds were surface-sterilized with 5% (*v*/*v*) sodium hypochlorite and rinsed three times with deionized water. Sterilized seeds were sown in experimental pools (1 m × 1 m × 0.8 m) filled with 350 kg of soil. Each treatment was replicated in three independent cultivation containers (pools), with each pool serving as a biological replicate. Within each pool, plants were treated as technical subsamples. All measurements from subsamples were averaged per pool prior to statistical analysis. A completely randomized design was used, and container positions were re-randomized weekly to minimize positional effects. All pools were filled with the same batch of homogenized topsoil, which was thoroughly mixed prior to distribution to ensure uniform initial physicochemical and biological properties across treatments. The baseline soil properties (e.g., pH, DOC) were measured for subsequent use in soil health assessment alongside the application of Nano-ZSe. Plants were grown in a greenhouse under a 20/15 °C (day/night) temperature regime and an 18/6 h (light/dark) photoperiod. The experiment included four foliar spray treatments: (1) control (CK), sprayed with distilled water only; (2) Nano-ZSe, a Se and Zn nanocomposite containing both Se ENMs and ionic Zn; (3) Se ENMs (0.18 mg·kg^−1^); and (4) Zn fertilizer (0.22 mg·kg^−1^). The CK treatment served as the baseline control for all comparisons. Treatments (3) and (4) were designed solely to evaluate the individual contributions of Se ENMs and ionic Zn, respectively, and their effects are interpreted exclusively in relation to the combined Nano-ZSe treatment (2), All dose rates are expressed as mg of active element (Se or Zn) per kg of soil in each pool. Each treatment was replicated in three independent pools. At 15 days after sowing, 150 g of the respective formulation was applied to each pool by foliar spraying. The cultivation period lasted 45 days from sowing to harvest. At harvest, *B. chinensis* plants were collected. Fresh leaf tissues were immediately frozen in liquid nitrogen and stored at −80 °C for metabolic and molecular analyses. Another portion of the samples was dried at 105 °C for 30 min and then at 60 °C to constant weight for nutrient analysis. Detailed procedures for metabolic profiling and gene expression analysis are described in Texts S1 and S2.

#### 2.2.2. Determination of *B. chinensis* Quality

Quality attributes of *B. chinensis* leaf samples stored at −80 °C were evaluated using commercially available assay kits according to the manufacturers’ instructions, with spectrophotometric quantification of all analytes. Protein concentration was determined by the biuret method (Solarbio Science & Technology Co., Ltd., Beijing, China). This assay relies on the formation of a violet-colored complex between peptide bonds and copper ions under alkaline conditions, with absorbance measured at 540 nm and directly proportional to protein concentration. Soluble sugar content was quantified using the anthrone-sulfuric acid method (Suzhou Grisis Biotechnology Co., Ltd., Suzhou, China). In this procedure, carbohydrates are dehydrated to form furfural derivatives, which subsequently condense with anthrone to yield a blue-green chromophore; absorbance was recorded at 620 nm for quantification. Total amino acid content was assessed via the ninhydrin reaction (Solarbio Science & Technology Co., Ltd., Beijing, China). α-Amino acids react with ninhydrin to produce Ruhemann’s purple, a purple chromophore exhibiting maximum absorbance at 570 nm. Vitamin C (ascorbic acid) content was determined using a kit from Shanghai Yeasen Biotechnology Co., Ltd., Shanghai (China). The method is based on the reduction of Fe^3+^ to Fe^2+^ by ascorbate under acidic conditions, followed by chelation of Fe^2+^ with 1,10-phenanthroline to form a red-orange complex. The absorbance of this complex at 534 nm is linearly correlated with ascorbate concentration. All assays were performed in triplicate.

#### 2.2.3. Determination of Nutrient Elements in *B. chinensis*

Dried leaf samples of *B. chinensis* were ground to a fine powder using a mortar and pestle. Approximately 25 mg of each powdered sample was accurately weighed into digestion tubes, with four replicate samples prepared for each treatment. To each tube, 3 mL of concentrated nitric acid (HNO_3_, guaranteed reagent grade) and 3 mL of ultrapure water (*v*/*v* = 1:1) were added. After pre-digestion at room temperature for 1 min, the samples were digested in a closed-vessel microwave system (Mars 6, CEM Corporation, Matthews, NC, USA) at 1400 W and 190 °C for 2 h. The resulting digestates were filtered through 0.22 μm membrane filters and quantitatively transferred to 50 mL volumetric flasks. The filtrates were diluted to the mark with ultrapure water. Concentrations of essential nutrient elements, including Se, Zn, phosphorus (P), potassium (K), magnesium (Mg), calcium (Ca), manganese (Mn), molybdenum (Mo), copper (Cu), and iron (Fe), were determined by inductively coupled plasma mass spectrometry (ICP-MS, ICAP TQ, Thermo Fisher, Waltham, MA, USA).

### 2.3. Soil Health Assessment via Comprehensive Index Determination

#### 2.3.1. Analysis of Soil Physicochemical and Microbial Indicators

To systematically assess the effects of exogenous amendments on soil health, a comprehensive set of physicochemical and microbial properties was evaluated. Soil moisture content (SMC) was determined gravimetrically by comparing the fresh weight to the oven-dried weight of soil samples, with three replicates per treatment. For measurement of pH, oxidation-reduction potential (ORP), and electrical conductivity (EC), air-dried soil samples were homogenized with deionized water at a soil-to-water ratio of 1:1.25 (*w*/*v*) and analyzed using a multiparameter meter (Mettler Toledo S400, Waltham, MA, USA). Microbial community composition was characterized using freshly collected soil samples stored at −80 °C. Bacterial and fungal community profiles were generated through high-throughput sequencing conducted by Magigene Biotechnology Co., Ltd. (Shenzhen, Guangdong, China). Detailed protocols for DNA extraction, amplification, sequencing, and bioinformatic analysis are provided in Text S3. To further evaluate soil nutrient availability and potential ecological implications, available phosphorus (AP), available potassium (AK), alkaline hydrolyzable nitrogen (AN), and soil organic carbon (SOC) were quantified. The analytical procedures for these indices are fully described in Text S4. 

#### 2.3.2. Soil Health Assessment

The reported soil health indicators represent post-treatment inter-treatment differences. A Minimum Data Set (MDS) was constructed using network analysis (NA) to identify key soil health indicators by elucidating complex interrelationships among soil properties, a method widely employed in soil microbial ecology [35]. Spearman correlation coefficients were calculated as input for network construction [36]. Nodes in the network represented individual soil indicators, while edges denoted significant correlations (R ≥ 0.60, *p* < 0.01). Network topology metrics were derived, with eigenvector centrality selected as a measure of node importance. This metric reflects not only the number of connections per node but also the relative significance of its neighboring nodes. The calculation formula is given as follows:

$$CE\left(N_{I}\right)=X_{i\;}=c\sum_{j=1}^{g}a_{i,j}x_{j}$$

where $X_{i}$ and $x_{j}$ represent the importance values of nodes *i* and *j*, respectively, *c* is a proportionality constant, *g* denotes the total number of nodes connected to node *i*, and $a_{i,j}$ = 1 if nodes *i* and *j* are significantly correlated, otherwise $a_{i,j}$ = 0. Indicators exhibiting high eigenvector centrality (i.e., within the top 10% of absolute values) were retained for further evaluation. In cases where multiple highly weighted indicators within a principal component demonstrated strong intercorrelations, the variable with the highest cumulative sum of correlation coefficients was selected for inclusion in the Soil Health Index (SHI) calculation [37].

For SHI computation, a methodology based on the area enclosed by a radar plot (SHI-area) was adopted [38]. This approach utilizes normalized values of the MDS indicators and does not involve subjective weighting or sequencing of variables. The normalization procedure is defined as:

$$stP_{i}=\frac{P_{i}}{P}$$

where $P_{i}$ represents the observed value of indicator *i* under a given treatment, and *P* denotes the reference value. For indicators where “lower is better” (i.e., lower values indicate higher functional performance), *P* corresponds to the minimum observed value. For indicators categorized as “best value” (i.e., the optimal value reflects maximum function), *P* represents the best (target) value [39].

$$SHI=0.5\times \sum_{i}^{n}stP_{i}^{2}\times \sin(\frac{2\times \pi }{n})$$

where *n* is the total number of selected indicators, and *π* is taken as 3.14.

### 2.4. Production and Economic Evaluation

Upon reaching physiological maturity, *B. chinensis* plants were harvested, and the above-ground shoot fresh weight was immediately measured. Crop yield was calculated as the shoot fresh weight per hectare. Economic benefit (*EB*) was estimated using the following formula:

EB=Yield×Price−Cost

where *EB* represents the net economic benefit in Chinese yuan (CNY), calculated as the annual crop yield (in kg·ha^−1^) multiplied by the market price per unit mass (in CNY·kg^−1^), minus the total production cost (in CNY·ha^−1^).

For economic evaluation, the 2024 market price of *B. chinensis* in Suzhou was adopted, considering that the experimental site was located within a greenhouse facility in Kunshan. The economic benefit was thus determined as the product of crop yield and the corresponding price in Chinese yuan, adjusted for total production costs.

### 2.5. Statistical Analysis

All treatments included at least three independent biological replicates. Data are expressed as mean ± standard deviation (SD). Differences among treatment groups were evaluated by one-way analysis of variance (ANOVA), followed by Fisher’s least significant difference (LSD) post hoc test for multiple comparisons. Statistical analyses were performed using OriginPro 2018 (OriginLab Corporation, Northampton, MA, USA), with statistical significance defined as *p* < 0.05.

## 3. Results and Discussion

### 3.1. Optimization of Foliar-Applied Nano-ZSe Concentration

The synthesized Nano-ZSe exhibited homogeneous spherical morphology with a mean hydrodynamic diameter of 41.5 ± 8.2 nm, as confirmed by TEM imaging and ImageJ-based particle size analysis (Figure S1a). High-resolution XPS spectra revealed distinct chemical states: the Se 3d peak centered at 55.3 eV corresponds to elemental selenium (Se^0^), confirming the successful reduction of SeO_2_ to nano-Se; meanwhile, the Zn characteristic binding energies at 990 eV indicates the presence of Zn^2+^ species, likely derived from the commercial Zn fertilizer used in formulation (Figure S1b). These results collectively demonstrate the successful preparation of a stable physical blend of elemental Se nanoparticles and Zn^2+^, consistent with the intended design.

**Figure 1:**
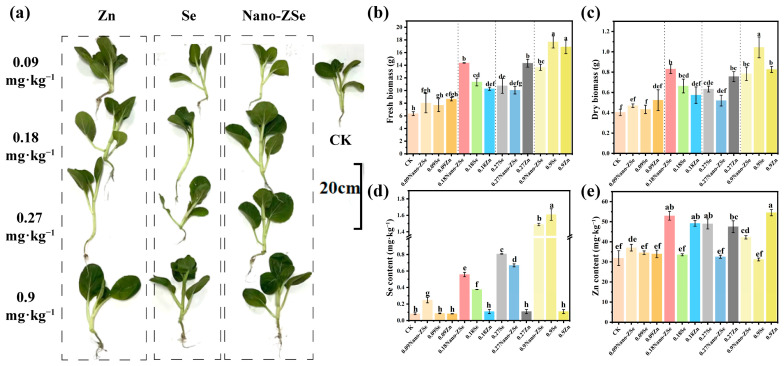
Biological effects of materials at different concentrations: Effect photos (**a**); Fresh weight (**b**); and Dry weight (**c**) of *B. chinensis*; Se (**d**) and (**e**) Zn content in *B. chinensis* leaves. Different letters indicate significant differences among treatments (*p* < 0.05, LSD, *n* = 3).

To identify the optimal foliar dosage that balances agronomic performance and nutritional safety, we evaluated a gradient of Nano-ZSe concentrations (0.09–0.9 mg·kg^−1^) on mature *B. chinensis* plants. Notably, the 0.18 mg·kg^−1^ treatment outperformed all others in promoting biomass accumulation, increasing fresh and dry weights by 126.5% and 107.5%, respectively, relative to the untreated control (CK) (Figure 1b,c). While higher doses (0.27 and 0.9 mg·kg^−1^) also stimulated growth (elevating fresh weight by 69.4% and 115.3%), they failed to match the efficacy of the 0.18 mg·kg^−1^ regimen, suggesting a non-linear dose–response relationship potentially linked to nutrient saturation or mild phytotoxicity at elevated levels.

Critically, the 0.18 mg·kg^−1^ dose also achieved the highest Zn bioaccumulation in edible tissues, with a 166% increase over CK, surpassing gains observed at 0.09 mg·kg^−1^ (116%), 0.27 mg·kg^−1^ (154%), and 0.9 mg·kg^−1^ (133%) (Figure 1e). In contrast, Se content exhibited a monotonic increase with dosage, rising 3.01-, 7.00-, 10.12-, and 17.62-fold across the same gradient (Figure 1d). Although all treatments exceeded the minimum threshold for Se-enriched produce (≥0.15 mg·kg^−1^ dry weight, GH/T 1135-2024; Selenium-rich agricultural products. All-China Supply and Marketing Cooperatives: Beijing, China, 2024), the 0.27 and 0.9 mg·kg^−1^ applications surpassed the upper safety limit (≤1.0 mg·kg^−1^), rendering them unsuitable for functional food applications due to potential human health risks. Collectively, the 0.18 mg·kg^−1^ Nano-ZSe treatment uniquely reconciles maximal biomass enhancement, peak Zn accumulation, and compliance with Se biofortification safety standards. This dosage was therefore selected for all subsequent mechanistic and field-scale investigations as the optimal balance between productivity, nutritional quality, and regulatory acceptability.

### 3.2. Nano-ZSe Modulates Stomatal Aperture via Metabolic Reprogramming

**Figure 2:**
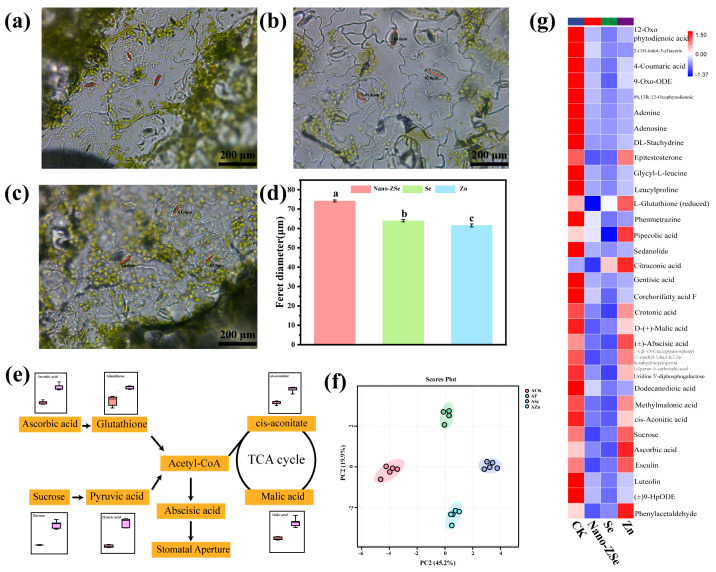
Metabolic changes in leaves: Photos of leaf stomata in the (**a**) Zn; (**b**) Nano-ZSe; and (**c**) Se ENMs treatment group; (**d**) Filit diameter; (**e**) Diagram of the metabolic mechanism of stomatal opening; (**f**) Diagram of principal component analysis of metabolism; (**g**) Metabolic heat map. Different letters indicate significant differences among treatments (*p* < 0.05, LSD, *n* = 3).

Foliar application of Nano-ZSe significantly enhanced Se and Zn accumulation in *Brassica chinensis* tissues compared to other treatments (Figure 1d,e). Given that stomata serve as primary entry points for ENMs in plant leaves [40,41,42], we hypothesized that stomatal infiltration is a dominant route for Nano-ZSe internalization. This premise is supported by prior studies demonstrating stomatal uptake of CeO_2_, Ag, and TiO_2_ nanoparticles following foliar exposure [43]. Consistent with this notion, Nano-ZSe application significantly increased stomatal aperture in *B. chinensis*, with the Feret diameter elevated by 15.91% and 20.53% relative to Se ENMs or Zn fertilizer alone, respectively (Figure 2a–d), indicating a synergistic effect of co-delivered Se and Zn on stomatal dynamics.

Stomatal movement is governed by a complex interplay of environmental cues, phytohormonal signals, and metabolic fluxes [44,45]. To elucidate the metabolic basis of Nano-ZSe-induced stomatal opening, untargeted metabolomic profiling was performed, leading to the identification and semi-quantification of 100 metabolites in leaf tissues. Principal component analysis (PCA) revealed clear separation among the four treatment groups along PC1 (45.2% variance) and PC2 (19.9% variance) (Figure 2f). Notably, Nano-ZSe treatment resulted in significant downregulation of several key metabolites compared to Zn fertilizer alone, including cis-aconitic acid, malic acid, sucrose, abscisic acid (ABA), L-glutathione, 12-oxo-phytodienoic acid, and ascorbic acid (Figure 2g). Among these, ABA is a well-established regulator of stomatal closure under stress conditions [45]. The tricarboxylic acid (TCA) cycle (Figure 2e), a central hub of energy and precursor metabolism, supplies carbon skeletons for hormone biosynthesis, with acetyl-CoA synthesis mechanistically linked to TCA cycle activity [46]. KEGG pathway enrichment analysis indicated that Nano-ZSe perturbs ascorbate and glutathione metabolism, attenuates acetyl-CoA production, and suppresses TCA cycle activity. Furthermore, the reduced ABA levels may be partly due to Zn from Nano-ZSe enhancing antioxidant activity (e.g., via Cu/Zn-SOD), which could lower ROS and thereby suppress ABA synthesis—a possibility consistent with our stomatal observations [47]. These coordinated metabolic shifts are consistent with reduced ABA biosynthesis. Given the well-established role of ABA in promoting stomatal closure, the observed increase in stomatal aperture is consistent with reduced ABA levels in Nano-ZSe–treated plants. While stomatal modulation by nutrients has been sporadically noted, the idea that a Se and Zn nanocomposite might enhance foliar nutrient uptake by actively reprogramming stomatal behavior through metabolic–hormonal crosstalk appears to have received little attention in prior biofortification literature, potentially representing an underexplored dimension of nano-fertilizer efficacy. The concurrent alterations in central metabolism (e.g., TCA cycle suppression), ABA content, and stomatal behavior suggest a potential association among these processes. Therefore, Nano-ZSe potentially induces stomatal opening through metabolic reprogramming that suppresses ABA accumulation. Consequently, this physiological response potentially facilitates enhanced foliar uptake of Se and Zn, establishing a key mechanism underlying the superior biofortification efficacy of Nano-ZSe.

### 3.3. Nano-ZSe Mediates Transcriptional Regulation of Se and Zn Homeostasis Genes

The pronounced accumulation of Se and Zn in *B. chinensis* tissues following Nano-ZSe application is underpinned by coordinated transcriptional activation of key genes governing micronutrient uptake and translocation. As shown in Figure S2, Nano-ZSe, along with Se ENMs and Zn fertilizer, significantly upregulated a suite of transporters relative to CK, including *BcATPs*, *BcGSH1*, *BcSultr1;1*, *BcSultr1;2*, *BcSultr2;1*, *BcZIP2*, *BcZIP3*, *BcZIP4*, and *BcZIP6*. This transcriptional response aligns with established roles of these genes: *BcZIP*2-6 have been identified as central regulators of Zn acquisition and distribution in *B. chinensi* [48], while *ATPs*, *GSH1*, and *SULTRs* are known to mediate the assimilation of S and its chemical analog Se [49]. Furthermore, Nano-ZSe elicited a markedly stronger induction of these transporter genes compared to either Se ENMs or Zn fertilizer applied alone. This enhanced transcriptional activation directly parallels the synergistic elevation of Se and Zn concentrations observed in leaf tissues (Figure 1d,e), suggesting that the nanocomposite format enables more effective signaling or nutrient sensing than ionic or single-element nanoforms. The convergence of Se and Zn homeostasis pathways is further supported by prior studies: co-application of Zn and Se has been shown to boost ascorbic acid, soluble sugars, and mineral content in strawberry [50], while combined foliar ZnSO_4_ and Na_2_SeO_3_ improved biofortification efficiency by modulating sulfur-amino acid metabolism in wheat [51].

Collectively, these results demonstrate that Nano-ZSe orchestrates a synergistic transcriptional program that simultaneously upregulates Se- and Zn-specific transport systems. By co-activating *SULTR*-mediated Se uptake and *ZIP*-driven Zn import, two pathways that typically operate independently, Nano-ZSe overcomes the limitations of single-nutrient fertilization. This dual-regulation mechanism provides a molecular foundation for the superior co-biofortification performance of the Se-Zn nanocomposite, positioning it as a strategic tool for multi-nutrient enrichment in leafy vegetables.

### 3.4. Cultivation of Se- and Zn-Enriched B. chinensis Under Field Conditions

**Figure 3:**
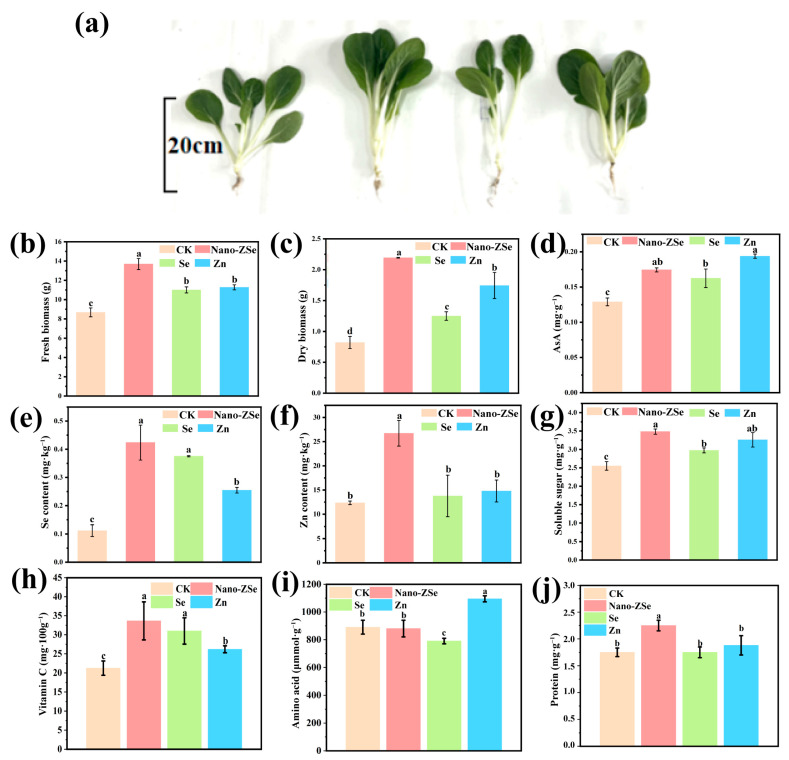
Biological effects of Nano-ZSe on *B. chinensis*: (**a**) Effect photos; (**b**) Fresh weight; (**c**) Dry weight; and (**d**) AsA content of *B. chinensis*; (**e**) Se; (**f**) Zn; (**g**) Soluble sugar content; (**h**) Vitamin C; (**i**) Amino acid; and (**j**) Protein in *B. chinensis* leaves. Different letters indicate significant differences among treatments (*p* < 0.05, LSD, *n* = 3).

This study demonstrates that foliar application of Nano-ZSe significantly enhances both the agronomic performance and nutritional quality of *B. chinensis* under field conditions. Relative to CK, Nano-ZSe treatment increased Se and Zn concentrations by 3.82- and 2.17-fold, respectively (Figure 3e,f). Notably, these values surpassed those achieved with individual Se ENMs or Zn fertilizer treatments by 13.51% and 80.78%, respectively, indicating an enhanced co-accumulation biofortification effect. Concurrently, shoot fresh and dry weights were elevated by 29.79% and 70.97% (Figure 3b,c), further demonstrating the superior growth-promoting capacity of Nano-ZSe compared to single-element treatments. Specifically, Nano-ZSe enhanced fresh weight by 12.31% relative to Se ENMs and by 16.56% relative to Zn fertilizer, while dry weight increased by 11.58% and 3.92%, respectively. The growth enhancement observed with Nano-ZSe may be attributed to the well-documented role of Se ENMs in improving photosynthetic efficiency [52]. However, the precise mechanistic interplay between Se and Zn remains an area for future investigation. Nonetheless, the synergistic effects on biomass accumulation underscore the potential of Nano-ZSe as a versatile tool for enhancing crop productivity. Beyond yield improvements, Nano-ZSe also markedly enhanced the nutritional profile of *B. chinensis*. Essential mineral concentrations, including Mg, P, S, Ca, and Fe, were significantly elevated (Figure S3), likely due to synergistic nutrient interactions. Additionally, key nutritional components such as amino acids, protein, soluble sugars, and vitamin C showed substantial increases relative to CK (Figure 3d–j). The observed rise in vitamin C content is consistent with prior reports linking Zn fertilization to antioxidant enhancement [53]. These nutrients play critical roles in human health, supporting physiological functions such as bone mineralization, energy metabolism, immune function, and oxidative stress mitigation [54,55].

Collectively, these findings establish Nano-ZSe as a highly effective strategy for dual Se-Zn biofortification, offering a practical solution to address micronutrient deficiencies in leafy vegetable production systems. By simultaneously boosting both elemental concentrations and overall nutritional quality, Nano-ZSe represents a promising approach for enhancing the nutritional value of crops, thereby contributing to improved public health outcomes. Importantly, the elevated selenium levels remain well within safe limits for human consumption. Based on the established exposure assessment framework (Supplementary Text S5), the Estimated Daily Intake (EDI) and Hazard Risk Index (HRI) were calculated as 1.1 × 10^−4^ mg·kg^−1^·day^−1^ and 0.022, respectively. Given that HRI ≪ 1, regular consumption of Nano-ZSe–treated *Brassica chinensis* is unlikely to pose any appreciable health risk, supporting its safety as a Se-biofortified functional food.

### 3.5. Area-Based Soil Health Assessment Under Nano-ZSe Exposure

**Figure 4:**
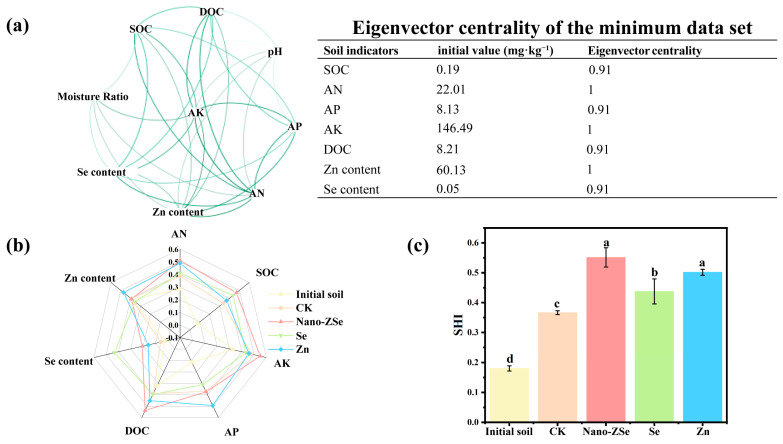
Soil health assessment: (**a**) Network analysis diagram; (**b**) Radar area diagram of soil health; (**c**) Soil health index. Different letters indicate significant differences among treatments (*p* < 0.05, LSD, *n* = 3).

Conventional fertilization strategies often neglect long-term soil health, yet sustainable crop production depends on the functional integrity of the soil ecosystem. To address this gap, we employed an area-based soil health index (SHI) approach, grounded in a minimum data set (MDS) of key indicators identified via Spearman correlation network analysis (eigenvector centrality > 0.9; Figure 4a). The resulting MDS included SOC, AK, DOC, AP, Se, Zn, and SMC which collectively represent chemical, biological, and physical dimensions of soil function. Among these, SOC was consistently retained as a core indicator due to its foundational role in soil formation, where it governs aggregate stability, nutrient cycling, water-holding capacity, and microbial habitat quality [56,57]. Intriguingly, the highest SOC level coincided with peak microbial diversity under Nano-ZSe treatment (Figure 4b), suggesting positive feedback between organic carbon accrual and microbial vitality. Quantitative SHI assessment revealed that Nano-ZSe significantly improved overall soil health, increasing the index by 54.84% relative to CK. Moreover, Nano-ZSe outperformed both Se ENMs and Zn fertilizer alone, enhancing SHI by 33.33% and 33.32%, respectively (Figure 4c), thereby demonstrating its superior capacity to sustain multifunctional soil processes.

We analyzed soil microbial community structure. Nano-ZSe application markedly elevated α-diversity relative to CK, with increases of 32.3% in Chao1, 35.8% in ACE, 25.9% in species richness, and 34.94% in the Shannon index (Figure S4). These metrics were also consistently higher than those observed under single-element treatments, confirming that the Se–Zn nanocomposite exerts the strongest stimulatory effect on microbial richness and evenness. Therefore, these findings reveal that Nano-ZSe transcends conventional nutrient delivery by concurrently enhancing crop performance, nutritional quality, and soil ecosystem functionality. By fostering a synergistic interplay among Se/Zn availability, organic carbon dynamics, and microbial diversity, Nano-ZSe establishes a plant–soil co-beneficial paradigm. This study thus provides a methodological and conceptual framework for next-generation fertilization strategies that integrate agronomic productivity with soil health stewardship. Although, it should be acknowledged that this study did not assess the long-term fate of Nano-ZSe components in soil systems. Critical questions remain regarding the persistence, transformation, and potential accumulation of Nano-ZSe following repeated applications, which could influence soil biogeochemistry, microbial functionality, or crop safety over time. While our short-term data show no adverse effects, the absence of multi-season or multi-year trials means that potential risks associated with nano-residue buildup cannot yet be ruled out. Therefore, comprehensive environmental risk assessments including long-term soil monitoring and ecotoxicological profiling are essential before widespread deployment.

### 3.6. Evaluation of Crop Yield and Economic Performance

Building on the biomass enhancements reported in Section 3.4, we conducted a field-scale evaluation of yield and economic performance to quantify the practical advantages of Nano-ZSe. Foliar application of Nano-ZSe substantially outperformed CK, increasing *B. chinensis* yield by 57.18% and net economic returns by 55.20% per hectare (Figure S5a,b). In comparison, Se ENMs and Zn fertilizer alone yielded more modest gains 26.85% and 29.93% in yield, and 24.95% and 29.19% in economic returns, respectively. Importantly, Nano-ZSe demonstrated clear superiority over single-element treatments: it further elevated yield by 24.41% relative to Se ENMs and by 21.47% relative to Zn fertilizer, with corresponding economic advantages of 24.12% and 20.13%. These results provide compelling evidence of a synergistic interaction between Se and Zn not only in physiological and nutritional outcomes, but also in agronomic productivity and financial viability.

From a practical farming perspective, adopting Nano-ZSe could translate into a 55.20% increase in net profit per hectare-a margin substantial enough to incentivize adoption among smallholder and commercial growers alike. Together with its demonstrated benefits for crop nutrition, soil health, and microbial diversity (Section 3.3, Section 3.4 and Section 3.5), Nano-ZSe emerges as a high-efficiency, economically viable, and environmentally conscious strategy for modern vegetable production. This integrated performance positions Nano-ZSe not merely as a fertilizer alternative, but as a scalable solution for advancing nutrient-dense, profitable, and sustainable agriculture. However, due to the limited replication (*n* = 3) and the absence of large-scale field implementation. Thus, while our results offer promising preliminary evidence for Nano-ZSe–mediated dual biofortification, validation in larger, more diverse trials is needed before broader application.

## 4. Conclusions

In this work, foliar application of Nano-ZSe effectively enabled dual biofortification of selenium and zinc in *B. chinensis*, achieving 3.82- and 2.17-fold higher accumulation in edible tissues compared to CK. This performance substantially exceeded that of single-element treatments (Se ENMs or Zn fertilizer alone), suggesting a coordinated enhancement between Se and Zn uptake at both physiological and molecular levels. The observed increase in nutrient uptake may be associated with metabolic reprogramming in leaves, where reduced activity of the TCA cycle appears to lower acetyl-CoA availability and downregulate ABA biosynthesis, potentially promoting stomatal opening and facilitating foliar absorption. Additionally, Nano-ZSe treatment was linked to the upregulation of key transporter genes (*BcSULTRs* and *BcZIPs*), which may contribute to enhanced translocation and co-accumulation of Se and Zn in shoots. Beyond nutritional enhancement, Nano-ZSe significantly improved agronomic outcomes: shoot dry biomass increased by up to 70.97%, field-scale yield rose by 57.18%, and net economic returns per hectare were elevated by 55.20% relative to CK. Moreover, the technology also showed potential to improve soil functionality, as indicated by a 54.84% improvement in the quantitative soil health index and significant enrichment of microbial diversity. These attributes suggest that Nano-ZSe could serve as a practical solution for addressing micronutrient deficiency (“hidden hunger”), enhancing farm profitability, and improving soil sustainability. However, it is important to note that these findings are based on greenhouse experiments using *B. chinensis*; further studies under diverse environmental conditions and across different crop species will be necessary to fully evaluate the efficacy and scalability of Nano-ZSe-enabled foliar biofortification strategies. While this study demonstrates the efficacy of foliar-applied Nano-ZSe for dual Se/Zn biofortification, the environmental fate of such nanomaterials warrants further attention. Rainfall or irrigation may wash nanoparticles from leaf surfaces into the soil, where their persistence, transformation, and potential interactions with soil microbiota remain unclear. Future work should therefore assess the ecotoxicological profile of Nano-ZSe residues, particularly their impact on soil microbial communities and nutrient cycling processes, to ensure sustainable deployment in agricultural systems.

## Data Availability

The original contributions presented in this study are included in the article/Supplementary Material. Further inquiries can be directed to the corresponding author.

## Supplementary Materials

The following supporting information can be downloaded at: https://www.mdpi.com/article/10.3390/nano16010056/s1, Text S1: Metabolic profiling in *B. chinensis* leaves; Text S2: Quantitative analysis of genes associated with Se and Zn uptake and translocation; Text S3: High-throughput sequencing-based characterization of soil microbial communities; Text S4: Assessment of soil fertility parameters; Text S5: Se risk analysis (EDI/HRI); Figure S1: Characterization diagram of Nano-ZSe; Figure S2: Heat map of the relative expression levels of genes related to Se and Zn absorption in leaves; Figure S3: Nutrient element concentration in *B. chinensis* leaves; Figure S4: α diversity indices of soil microorganisms; Figure S5: (a) Yield; and (b) Economic benefits of *B. chinensis* per hectare; Table S1: Primer sequences used in this study. References [58,59] are included in the Supplementary Materials.

## References

[1] Burchi F., Fanzo J., Frison E. (2011). The Role of Food and Nutrition System Approaches in Tackling Hidden Hunger. Int. J. Environ. Res. Public Health.

[2] Hong T., Zhao Z., Bian W., Zhu W., Li Z., Shen G., Gu Y., Chen L., Guo Y. (2023). Development of a novel nutritional assessment model based on strontium and other compositional factors in apples across seven regions in China. Front. Sustain. Food Syst..

[3] Roriz M., Carvalho S.M., Castro P.M., Vasconcelos M.W. (2020). Legume Biofortification and the Role of Plant Growth-Promoting Bacteria in a Sustainable Agricultural Era. Agronomy.

[4] Zhao B., Ding H., Hu T., Guo Y. (2023). Synergistic effects of the Se and Zn supplemental combination on the nutrient improvement of mannitol and adenosine and the multi-element bioaccessibility in *Cordyceps cicadae*. LWT-Food Sci. Technol..

[5] Sunic K., Spanic V. (2024). Genetic biofortification of winter wheat with selenium (Se). Plants.

[6] Jones G.D., Droz B., Greve P., Gottschalk P., Poffet D., McGrath S.P., Seneviratne S.I., Smith P., Winkel L.H. (2017). Selenium deficiency risk predicted to increase under future climate change. Proc. Natl. Acad. Sci. USA.

[7] Noulas C., Tziouvalekas M., Karyotis T. (2018). Zinc in soils, water and food crops. J. Trace Elem. Med. Biol..

[8] Natasha N., Shahid M., Bibi I., Iqbal J., Khalid S., Murtaza B., Bakhat H.-F., Farooq A.U., Amjad M., Hammad H.-M. (2022). Zinc in soil-plant-human system: A data-analysis review. Sci. Total Environ..

[9] Jing M.Y., Sun J.Y., Weng X.Y. (2007). Insights on zinc regulation of food intake and macronutrient selection. Biol. Trace Elem. Res..

[10] Oztekin Y., Buyuktuncer Z. (2025). Agronomic biofortification of plants with iodine and selenium: A potential solution for iodine and selenium deficiencies. Biol. Trace Elem. Res..

[11] Kong L., Tao Y., Xu Y., Zhou X., Fu G., Zhao L., Wang Q., Li H., Wan Y. (2024). Simultaneous biofortification: Interaction between zinc and selenium regarding their accumulation in wheat. Agronomy.

[12] Zhang H., Mi K., Chen J., Cui P., Lu H., Zhang H., Yang Y. (2025). Enhancing rice yield, quality and nitrogen utilization through side-deep placement of nitrogen and zinc fertilizers. Field Crops Res..

[13] Cheng B., Wang C., Yue L., Chen F., Cao X., Lan Q., Liu T., Wang Z. (2023). Selenium nanomaterials improve the quality of lettuce (*Lactuca sativa* L.) by modulating root growth, nutrient availability, and photosynthesis. NanoImpact.

[14] Cheng B., Wang C., Chen F., Yue L., Cao X., Liu X., Yao Y., Wang Z., Xing B. (2022). Multiomics understanding of improved quality in cherry radish (*Raphanus sativus* L. *var. radculus pers*) after foliar application of selenium nanomaterials. Sci. Total Environ..

[15] Guelfi D., Nunes A.P.P., Sarkis L.F., Oliveira D.P. (2022). Innovative phosphate fertilizer technologies to improve phosphorus use efficiency in agriculture. Sustainability.

[16] Babu R.S., Joseph M., Hemalatha M., Bhuvaneswari J., Srinivasan S., Leninraja D. (2024). Nano-fertilizers: The future of nutrient approaches for cereals. Indian J. Agric. Sci..

[17] Hadri S.H., Afzaal A., Saeed L., Arshad A., Nazeer S., Akram M. (2024). Recent advances in the development of nanoparticle based fertilizers for different kinds of crops: A review. Biocatal. Agric. Biotechnol..

[18] Kekeli M.A., Wang Q., Rui Y. (2025). The role of nano-fertilizers in sustainable agriculture: Boosting crop yields and enhancing quality. Plants.

[19] Gupta P., Dhar H., Bagal Y.S., Jaglan S. (2025). Smart nano-fertilizers: A path to sustainable agriculture. Environ. Geochem. Health.

[20] Goyal A., Chavan S.S., Mohite R.A., Shaikh I.A., Chendake Y., Mohite D.D. (2025). Emerging trends and perspectives on nano-fertilizers for sustainable agriculture. Discov. Nano.

[21] Wang Z., Yue L., Dhankher O.P., Xing B. (2020). Nano-enabled improvements of growth and nutritional quality in food plants driven by rhizosphere processes. Environ. Int..

[22] Wang C., Cheng B., Li J., Li X., Feng Y., Kah M., Yue L., Cao X., Fan Z., Ji Y. (2025). Application of selenium-engineered nanomaterials to paddy soil promote rice production by improving soil health. Commun. Earth Environ..

[23] Lei C., Ding Z., Tao M., Lu Y., Xu L., Cheng B., Wang C., Wang Z. (2024). Unraveling the distribution, metabolization, and catabolism of foliar sprayed carbon dots in maize and effect on soil environment. J. Agric. Food Chem..

[24] Ren Y., Zhang D., Cheng B., Chen B., Yue L., Cao X., Wang Z., Wang Z. (2025). Foliar Spraying Zinc–Carbon Dot Nanofertilizer Promotes Yield and Quality of Lettuce (*Lactuca sativa* L.) through Leaf–Root Regulation. ACS Agric. Sci. Technol..

[25] Kałucka M., Podsadni P., Szczepańska A., Malinowska E., Błażewicz A., Turło J. (2025). Impact of Interactions Between Zn (II) and Selenites in an Aquatic Environment on the Accumulation of Se and Zn in a Fungal Cell. Molecules.

[26] Wang X., Hussain B., Xin X., Zou T., Huang X., Cheng L., Wu Z., Yang Y., Li Y., He Z. (2025). Fate and Physiological Effects of Foliar Selenium Nanoparticles in Wheat. ACS Nano.

[27] Cheng B., Liu J., Li X., Yue L., Cao X., Li J., Wang C., Wang Z. (2024). Bioavailability of selenium nanoparticles in soil and plant: The role of particle size. Environ. Exp. Bot..

[28] El-Ramady H., Faizy S.E.D., Abdalla N., Taha H., Domokos-Szabolcsy É., Fari M., Elsakhawy T., Omara A.E., Shalaby T., Bayoumi Y. (2020). Selenium and nano-selenium biofortification for human health: Opportunities and challenges. Soil Syst..

[29] Burmistrov D.E., Shumeyko S.A., Semenova N.A., Dorokhov A.S., Gudkov S.V. (2025). Selenium Nanoparticles (Se NPs) as Agents for Agriculture Crops with Multiple Activity: A Review. Agronomy.

[30] Samynathan R., Venkidasamy B., Ramya K., Muthuramalingam P., Shin H., Kumari P.S., Thangavel S., Sivanesan I. (2023). A recent update on the impact of nano-selenium on plant growth, metabolism, and stress tolerance. Plants.

[31] Zhou C., Miao P., Xu Z., Yi X., Yin X., Li D., Pan C. (2024). Exploring the mechanism of nano-selenium treatment on the nutritional quality and resistance in plum plants. Ecotox. Environ. Safe.

[32] Zahedi S.M., Hosseini M.S., Meybodi N.D.H., Silva J.A.T. (2019). Foliar application of selenium and nano-selenium affects pomegranate (*Punica granatum* cv. Malase Saveh) fruit yield and quality. S. Afr. J. Bot..

[33] Zhu Y., Dong Y., Zhu N., Jin H. (2022). Foliar application of biosynthetic nano-selenium alleviates the toxicity of Cd, Pb, and Hg in Brassica chinensis by inhibiting heavy metal adsorption and improving antioxidant system in plant. Ecotox. Environ. Safe.

[34] Zhou X., Yang J., Kronzucker H.J., Shi W. (2020). Selenium biofortification and interaction with other elements in plants: A review. Front. Plant Sci..

[35] Jiao S., Lu Y., Wei G. (2022). Soil multitrophic network complexity enhances the link between biodiversity and multifunctionality in agricultural systems. Glob. Change Biol..

[36] Raiesi F., Beheshti A. (2022). Evaluating forest soil quality after deforestation and loss of ecosystem services using network analysis and factor analysis techniques. Catena.

[37] Li P., Wu M., Kang G., Zhu B., Li H., Hu F., Jiao J. (2020). Soil quality response to organic amendments on dryland red soil in subtropical China. Geoderma.

[38] Kuzyakov Y., Gunina A., Zamanian K., Tian J., Luo Y., Xu X., Yudina A., Aponte H., Alharbi H., Ovsepyan L. (2020). New approaches for evaluation of soil health, sensitivity and resistance to degradation. Front. Agric. Sci. Eng..

[39] Yuan P., Wang J., Li C., Xiao Q., Liu Q., Sun Z., Wang J., Cao C. (2020). Soil quality indicators of integrated rice-crayfish farming in the Jianghan Plain, China using a minimum data set. Soil Tillage Res..

[40] Avellan A., Yun J., Morais B.P., Clement E.T., Rodrigues S.M., Lowry G.V. (2021). Critical review: Role of inorganic nanoparticle properties on their foliar uptake and in planta translocation. Food Energy Secur..

[41] Gautam K., Singh H., Sinha A.K. (2025). Nanotechnology in Plant Nanobionics: Mechanisms, Applications, and Future Perspectives. Adv. Biol..

[42] Hu P., An J., Faulkner M.M., Wu H., Li Z., Tian X., Giraldo J.P. (2020). Nanoparticle charge and size control foliar delivery efficiency to plant cells and organelles. ACS Nano.

[43] Hong J., Peralta-Videa J.R., Rico C., Sahi S., Viveros M.N., Bartonjo J., Zhao L., Gardea-Torresdey J.L. (2014). Evidence of translocation and physiological impacts of foliar applied CeO_2_ nanoparticles on cucumber (*Cucumis sativus*) plants. Environ. Sci. Technol..

[44] Singh A., Roychoudhury A. (2023). Abscisic acid in plants under abiotic stress: Crosstalk with major phytohormones. Plant Cell Rep..

[45] Ahmad S., Belwal V., Punia S.S., Ram M., Dalip, Rajput S.S., Kunwar R., Meena M., Gupta D., Kumawat G. (2023). Role of plant secondary metabolites and phytohormones in drought tolerance: A review. Gesunde Pflanz..

[46] Feng W., Yuan J., Gao F., Weng B., Hu W., Lei Y., Huang X., Yang L., Shen J., Zhang S. (2020). Piezopotential-driven simulated electrocatalytic nanosystem of ultrasmall MoC quantum dots encapsulated in ultrathin N-doped graphene vesicles for superhigh H_2_ production from pure water. Nano Energy.

[47] Hernández J., Díaz-Vivancos P., Acosta-Motos J., Alburquerque N., Martínez D., Carrera E., García-Bruntón J., Barba-Espín G. (2021). Interplay among antioxidant system, hormone profile and carbohydrate metabolism during bud dormancy breaking in a high-chill peach variety. Antioxidants.

[48] Wu X., Chen J., Yue X., Wei X., Zou J., Chen Y., Su N., Cui J. (2019). The zinc-regulated protein (ZIP) family genes and glutathione s-transferase (GST) family genes play roles in Cd resistance and accumulation of pak choi (*Brassica campestris* ssp. *chinensis*). Ecotoxicol. Environ. Safe.

[49] Zhang H., Hao X., Zhang J., Wang L., Wang Y., Li N., Guo L., Ren H., Zeng J. (2022). Genome-wide identification of SULTR genes in tea plant and analysis of their expression in response to sulfur and selenium. Protoplasma.

[50] Huang S., Gao L., Fu G., Du S., Wang Q., Li H., Wan Y. (2023). Interactive effects between zinc and selenium on mineral element accumulation and fruit quality of strawberry. Agronomy.

[51] Ning P., Fei P., Wu T., Li Y., Qu C., Li Y., Shi J., Tian X. (2022). Combined foliar application of zinc sulphate and selenite affects the magnitude of selenium biofortification in wheat (*Triticum aestivum* L.). Food Energy Secur..

[52] Wang C., Yue L., Cheng B., Chen F., Zhao X., Wang Z., Xing B. (2022). Mechanisms of growth-promotion and Se-enrichment in *Brassica chinensis* L. by selenium nanomaterials: Beneficial rhizosphere microorganisms, nutrient availability, and photosynthesis. Environ. Sci. Nano.

[53] Rabbi R.H.M., Chowdhury M.A.H., Uddin M.K., Saha B.K. (2024). Agronomic biofortification of zinc in tomato. J. Plant Nutr..

[54] Calvez J., Azzout-Marniche D., Tomé D. (2024). Protein quality, nutrition and health. Front. Nutr..

[55] Fang J., Yang Q., Maas R., Buono M., Meijlink B., Lotgerink Bruinenberg D., Benavente E., Mokry M., Mil A., Qian L. (2024). Vitamin C facilitates direct cardiac reprogramming by inhibiting reactive oxygen species. Stem Cell Res. Ther..

[56] Feifel M., Durner W., Hohenbrink T., Peters A. (2024). Effects of improved water retention by increased soil organic matter on the water balance of arable soils: A numerical analysis. Vadose Zone J..

[57] Liang Y., Fu R., Sailike A., Hao H., Yu Z., Wang R., Peng N., Li S., Zhang W., Liu Y. (2025). Soil labile organic carbon and nitrate nitrogen are the main factors driving carbon-fixing pathways during vegetation restoration in the Loess Plateau, China. Agric. Ecosyst. Environ..

[58] Pedron T., Augusto C., Silva G., Valeriano M., Mamián-López M., Slaveykova V., Batista B. (2025). Human health risk assessment and concentration of Al, Mn, Fe, Cu, Zn, Se, As, Cd, Pb, Hg, Rb, and REEs in chocolate. Food Chem. Toxicol..

[59] Hoque M., Tamanna F., Hasan M., Al Banna M., Mondal P., Prodhan M., Rahman M., van Brakel M. (2022). Probabilistic public health risks associated with pesticides and heavy metal exposure through consumption of common dried fish in coastal regions of Bangladesh. Environ. Sci. Pollut. Res..

