# Supplementary material for: Foliar-Applied Selenium–Zinc Nanocomposite Drives Synergistic Effects on Se/Zn Accumulation in Brassica chinensis L
Source: Nanomaterials (Basel). 2025 Dec 31;16(1):56. doi: 10.3390/nano16010056 (PMC12788064; doi:10.3390/nano16010056)
Supplement: Supplementary file 1 [file nanomaterials-16-00056-s001.zip › nanomaterials-4080068-supplementary.pdf]

Supporting Information

Foliar-applied selenium-zinc nanocomposite Drives Synergistic Effects on Se/Zn Accumulation in *Brassica chinensis* L

Mengna Tao, [#] Yusong Yao, [#] Lian Zhang, Jie Zeng, Bingxu Cheng, and Chuanxi Wang*

Institute of Environmental Processes and Pollution Control, and School of Environment and Ecology, Jiangnan University, Wuxi, Jiangsu 214122, China.

*Corresponding author:

Email address: wangcx2018@jiangnan.edu.cn (Dr. Chuanxi Wang)

Text S1. Metabolic profiling in *B. chinensis* leaves.

Leaf tissues from *B. chinensis* plants were harvested and immediately flash-frozen in liquid nitrogen to minimize metabolic alterations prior to analysis. Samples were stored at -80 °C until processing. Frozen tissues were mechanically disrupted under cryogenic conditions using a pre-cooled mortar and pestle, and the resulting powder was transferred into 2-mL microcentrifuge tubes (n=4 per treatment). Metabolites were extracted by adding 1.5 mL of pre-chilled methanol/water (80:20, v/v) to each sample, followed by vigorous vortex mixing. The mixtures were subjected to ultrasound-assisted extraction on ice at 35 kHz for 30 min, then centrifuged at 12,000×g and 4 °C for 15 min to remove insoluble debris. Supernatants were collected and concentrated to dryness under reduced pressure using a vacuum centrifuge equipped with a cold trap. The dried residues were reconstituted in 200 μL of methanol/acetonitrile/water (4:4:2, v/v/v), filtered through a 0.22 μm nylon membrane, and centrifuged again at 12,000×g and 4 °C for 10 min. The final extracts were analyzed by high-performance liquid chromatography coupled with tandem mass spectrometry (HPLC-MS/MS; Thermo, Germany) for untargeted metabolomic profiling.

Text S2. Quantitative analysis of genes associated with Se and Zn uptake and translocation

Total RNA was isolated from leaf tissues using a commercial plant RNA extraction kit (Titan, China), with on-column DNase I digestion applied to eliminate genomic DNA contamination. RNA integrity was verified by agarose gel

electrophoresis, and RNA concentration and purity were determined spectrophotometrically (NanoDrop One, Thermo Fisher). Complementary DNA (cDNA) was synthesized from 1 μg of total RNA using an EASY Reverse Transcription PreMix (Kangwei, China) according to the manufacturer's instructions, with a reaction mixture composed of RNA, pre-mix, and nuclease-free water in a 1:1:3 ratio. The expression levels of *BcATPs*, *BcGSH1*, *BcSultr1;1*, *BcSultr1;2*, *BcSultr2;1*, *BcZIP2*, *BcZIP3*, *BcZIP4*, and *BcZIP6*, putative transporters involved in Se and Zn homeostasis, were quantified via quantitative real-time PCR (qRT-PCR) using a CFX96™ Real-Time PCR Detection System (Bio-Rad, USA). Reactions were performed in 20 μL volumes with TransStart® Top Green qPCR SuperMix (TransGen Biotech, China) under the following thermal cycling conditions: 95 °C for 10 min; 40 cycles of 95 °C for 15 s and 60 °C for 60 s; followed by a melt curve analysis from 65 °C to 95 °C (0.5 °C increment every 5 s) to confirm amplification specificity. Each biological replicate was analyzed in quadruplicate technical replicates. Relative transcript abundance was calculated using the $2^{-\Delta\Delta CT}$ method, with *BcACTIN* and *BcUBQ* used as reference genes for normalization. Primer sequences are listed in Table S1.

Text S3. High-throughput sequencing-based characterization of soil microbial communities.

Soil microbial community composition was assessed by amplicon sequencing of taxonomically informative marker genes. Total genomic DNA was extracted from 0.5 g of homogenized soil using a magnetic bead-based DNA isolation kit (FINDROP,

China). DNA quality was evaluated by 1% agarose gel electrophoresis, and DNA concentration and purity (A260/A280 and A260/A230) were measured using a NanoDrop One spectrophotometer (Thermo Fisher Scientific). For fungal community analysis, the ITS1 region was amplified using primers 338F (5′-GGAAGTAAAAGTCGTAACAAGG-3′) and 806R (5′-GCTGCGTTCTTCATCGATGC-3′). For bacterial community profiling, the V3-V4 region of the 16S rRNA gene was amplified using primers 338F (5′-ACTCCTACGGGAGGCAGCA-3′) and 806R (5′-GGACTACHVGGGTWTCTAAT-3′). PCR reactions (25 μL) contained 50-200 ng template DNA, 1 μL of each primer (10 μM), 12.5 μL of 2× Premix Taq, and nuclease-free water. Thermal cycling conditions were as follows: initial denaturation at 94 °C for 5 min; 30 cycles of 94 °C for 30 s, 52 °C for 30 s, and 72 °C for 30 s; final extension at 72 °C for 5 min. Amplified products were purified using the E.Z.N.A.® Gel Extraction Kit (Omega Bio-tek), eluted in TE buffer, and normalized prior to library construction with the NEB Next® Ultra™ DNA Library Prep Kit (Illumina®). Indexed libraries were pooled and sequenced on an Illumina HiSeq or MiSeq platform (2 × 250 bp paired-end reads) at a commercial sequencing facility.

Text S4. Assessment of soil fertility parameters.

Key soil fertility parameters were determined using standardized analytical methods. AP was quantified by molybdenum-blue colorimetry: air-dried soil samples were extracted with sodium bicarbonate solution, and the filtrate was reacted with an antimony-potassium tartrate-molybdate reagent. The mixture was reduced with

ascorbic acid to form a phosphomolybdenum blue complex, and absorbance was measured at 700 nm. AN was determined using the alkaline hydrolysis-diffusion method; soil was incubated with 1.0 M NaOH in a diffusion chamber, and the released NH_3 was trapped in 2% boric acid and titrated with 0.01 M H_2SO_4. SOC was measured by the potassium dichromate oxidation method: organic matter was oxidized with $K_2Cr_2O_7$ under heating, and excess $Cr_2O_7^{2-}$ was back-titrated with ferrous sulfate. SOC content was calculated based on oxygen consumption and quantified at 585 nm. AK was extracted with 1 M ammonium acetate (pH=7.0) and determined turbidimetrically at 420 nm after reaction with sodium tetraphenylboron, which forms a colloidal precipitate proportional to K^+ concentration.

Text S5. Se risk analysis (EDI/HRI).

The estimated daily intake (EDI) was calculated according to the following equation [1]:

$$EDI = (C \times M) / BW$$

Where where C ($mg \cdot kg^{-1}$) is the (highest) concentration of a given element in *B. chinensis*, M ($kg \cdot day^{-1}$) is the average *B. chinensis*, consumption of 0.15 $kg \cdot day^{-1}$, and BW is the body mass (70 kg) [1].

Using the oral reference dose (RfD) for selenium of 5.0×10^{-3} $mg \cdot kg^{-1} \cdot day^{-1}$ (US EPA, 2004), the Hazard Risk Index (HRI) was calculated according to the following equation [2]:

$$HRI = EDI / RfD$$

Table S1. Primer sequences used in this study.

Primer name	Sequence (5'-3')
Actin-F	TACAACGAGCTCCGTGTTG
Actin-R	CATACGGTCAGCAATTCCAG
BcZIP2-F	CGGGATCCATGTCTTTCTCTTCCAAAACC
BcZIP2-R	AAGGAAAAAAGCGGCCGCTTAATCCCAAATCATAACAAT
BcZIP3-F	GGCATGTAGAGACGGAGACG
BcZIP3-R	CGGAGATTCCGAGAACGCTT
BcZIP4-F	CTTGGCGGGGATTTCTCCTT
BcZIP4-R	GAAAGTCTCGTTGGCATCGC
BcZIP6-F	TTCGAGTCTCTCGCCGATTG
BcZIP6-R	CACTCCGATCAACGTCACCA
BcATPs-F	TCGGAGGGTTCATGAGAGAG
BcATPs-R	GATCTTCCTTGGGATGCTTG
BcGSH1-F	GTTTCGTGCTGGTCTTGC
BcGSH1-R	GCGGTCCTTGTCAGTGTCT
BcSultr1;1-F	TCATGCACTCGGTATTCGGG
BcSultr1;1-R	CAACTGCCGGAACCCAAAAG
BcSultr1;2-F	ACTCTCCTTCGAGCTGAGGT
BcSultr1;2-F	AGCTGCTGAAGAGCGATTGT
BcSultr2;1-F	CGAGCTGTCTGGAGATCTTG
BcSultr2;1-R	TCGAGTCTGTACCCTTTTATTCCGGCGAACG

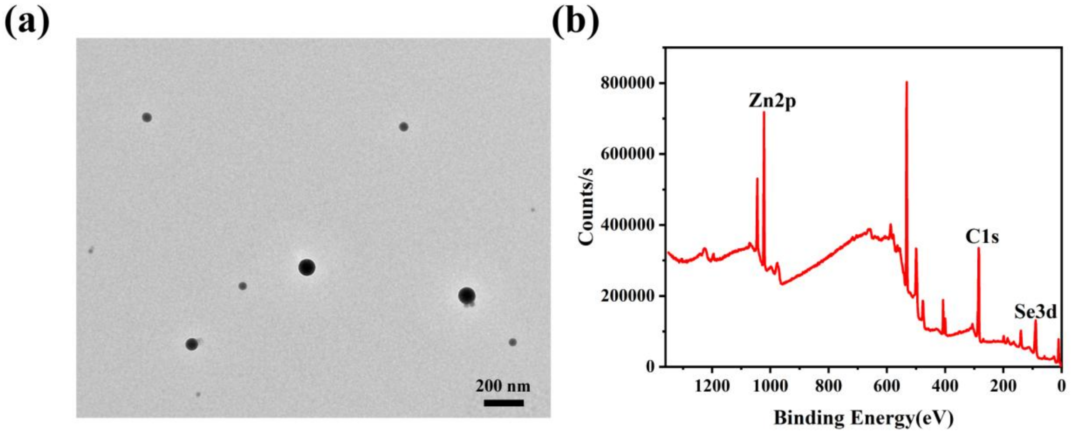

Figure S1. Characterization diagram of Nano-ZSe： (a) TEM; (b) XPS

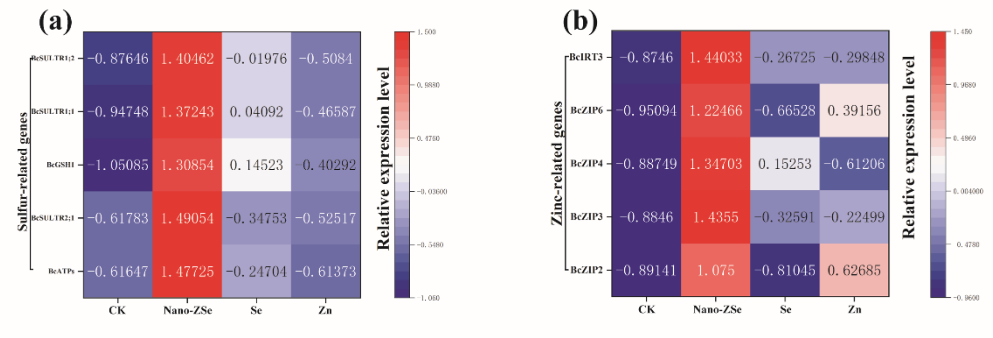

Figure S2. Heat map of the relative expression levels of genes related to Se and Zn absorption in leaves: Expression levels of genes related to (a) Se and (b) Zn absorption

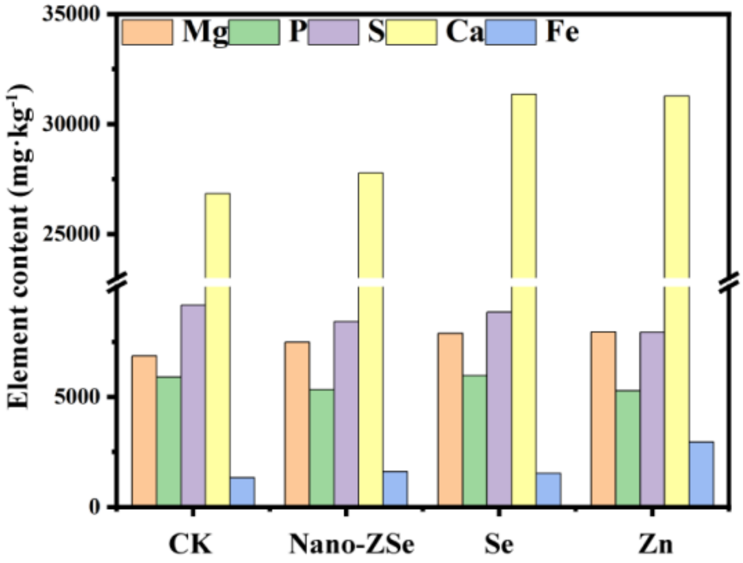

Figure S3. Nutrient element concentration in *B. chinensis* leaves.

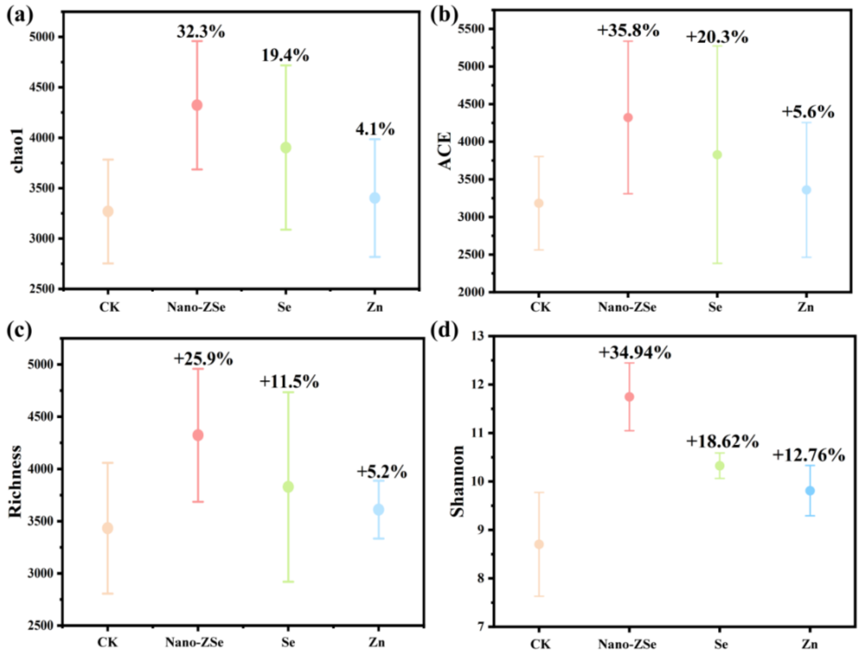

Figure S4. α diversity indices of soil microorganisms: (a) Chao1 index; (b) ACE index; (c) Richness index; (d)Shannon index

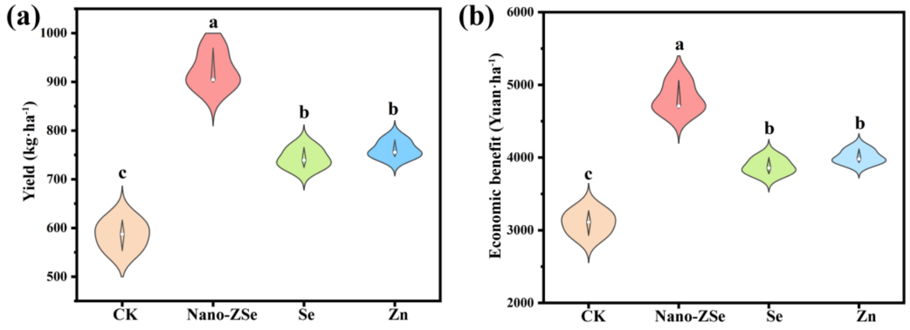

Figure S5. (a) Yield; and (b) Economic benefits of *B. chinensis* per hectare

References:

1. Pedron, T.; Augusto, C.; Silva, G.; Valeriano, M.; Mamián-López, M.; Slaveykova, V.; Batista, B. Human health risk assessment and concentration of Al, Mn, Fe, Cu, Zn, Se, As, Cd, Pb, Hg, Rb, and REEs in chocolate. *Food Chem. Toxicol*, **2025**, 115770.
2. Hoque, M.; Tamanna, F.; Hasan, M.; Al Banna, M.; Mondal, P.; Prodhan, M.; Rahman, M.; van Brakel, M. Probabilistic public health risks associated with pesticides and heavy metal exposure through consumption of common dried fish in coastal regions of Bangladesh. *Environ. Sci. Pollut. Res.* **2022**, *29*, 20112-20127.
